# The dual role of biochar in modulating microplastic effects on soil nitrogen

**DOI:** 10.3389/fmicb.2026.1862201

**Published:** 2026-09-07

**Authors:** Yanyan Zhang, Khalid Hussain, Xiaoyan Tang, Ting Lan, Chaorui Yan, Yang Li, Yingjie Wu, Jingjun Li, Xuesong Gao

**Affiliations:** 1 College of Resource, Sichuan Agriculture University, Chengdu, China; 2 Key Laboratory of Investigation and Monitoring, Protection and Utilization for Cultivated Land Resources, Ministry of Natural Resources, Chengdu, China; 3 International Science and Technology Cooperation Base for Efficient Utilization of Nutrient Resources and Fertilizer Innovation, Sichuan Provincial Department of Science and Technology, Chengdu, China; 4 State Key Laboratory of Simulation and Regulation of Water Cycle in River Basin, China Institute of Water Resources and Hydropower Research, Beijing, China

**Keywords:** biochar, inorganic nitrogen, microbial community, microplastics, particle size

## Abstract

Microplastics persist in the environment due to their resistance to degradation and pose an increasing concern for terrestrial ecosystems, threatening soil physicochemical properties and nitrogen (N) cycling. Biochar is widely used to improve soil quality and mitigate contamination. However little is known about how biochar modulates microplastic effects on soil N cycling. Therefore, we conducted a soil incubation experiment to investigate the effects of different polyethylene microplastic particle sizes (6.5, 600 and 1700 μm) on soil N dynamics and to evaluate the combined influence of biochar in microplastic-contaminated soils. Although significant differences among individual microplastic-size treatments were observed at some sampling times, no consistent particle-size dependent effect on soil nitrogen pools was observed throughout the incubation period. However, the addition of biochar significantly increased soil total N, nitrate-N, and alkali-hydrolyzable N contents. Microbial community analysis revealed that the 1700 μm polyethylene microplastic combined with biochar resulted in distinct microbial community structures compared with the control treatment, particularly after 45 days of incubation. Microbial community analysis revealed that the combination of polyethylene microplastics and biochar altered the composition of the dominant bacterial genera, with the greatest community shift observed under the 1700 μm microplastic treatment after 45 days of incubation. Overall, both microplastic presence and biochar amendment altered soil microbial community composition. These findings suggest that corn straw biochar may improve soil nitrogen availability under the experimental conditions of this study and provide insight into microbial responses to polyethylene microplastics. Additional studies across different soil types, biochar feedstocks, microplastic polymers, and field conditions are needed to evaluate the broader applicability of these findings.

## Graphical abstract

**Figure:**
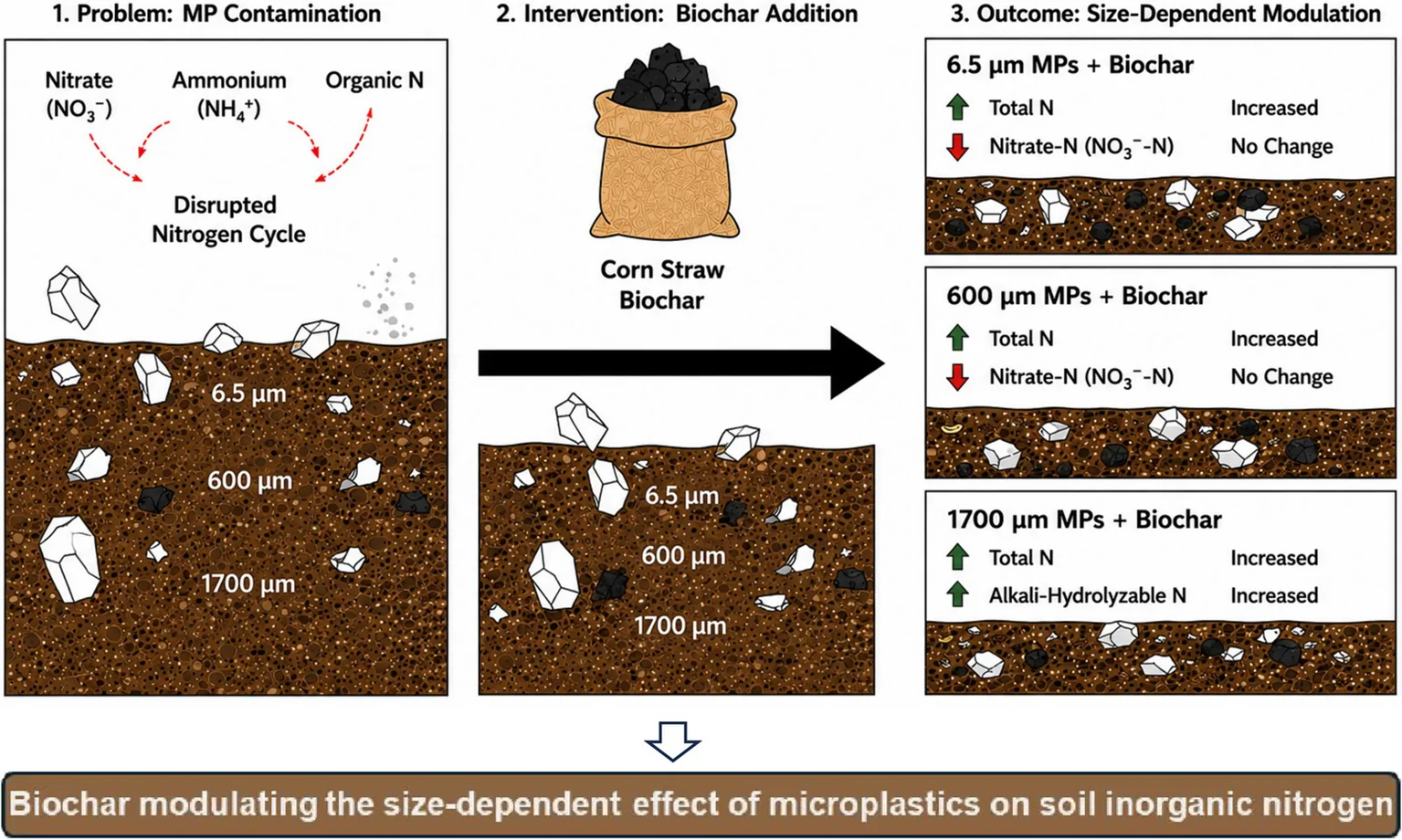

## 1 Introduction

Global plastic production is predicted to reach 34 billion tons by 2050. Currently, 32% of plastic are not adequately treated before being discarded into the environment (Ren et al., 2021; Sun et al., 2022). Microplastics (MPs) are particles smaller than 5 mm that are fragmented from plastic products (e.g., plastic mulching film, plastic pipes, greenhouse, etc.) (Xu et al., 2019). Abrasion, weathering, ultraviolet radiation, and microbial metabolism breaks plastics into microplastics, which accumulate into soil, water, and other environments over time (Zhang et al., 2020). This has resulted in microplastics accumulating in soil, water, and other areas over time. Due to their resistance to degradation, plastics potentially threaten ecosystems. Studies have found microplastics in agricultural soil at concentrations of up to 0.69 million particles per kilogram. Microplastics can enter the soil through different pathways such as plastic mulching, compost, sludge, coated fertilizer applications, sewage irrigation, and atmospheric deposition (Liu et al., 2023; Weithmann et al., 2018). Plastic mulching residue, particularly polyethylene (PE) mulching film, is a major contributor to microplastic pollution in agricultural soil (Rong et al., 2021). Microplastics have been shown to have a large specific surface area, numerous surface functional groups, and strong sorption capacity, all of which can impact soil physicochemical and microbial properties (Wang et al., 2021).

Microplastics significantly impact the physical and chemical properties of soil. Numerous studies have demonstrated that microplastics can alter key soil characteristics, including bulk density, porosity, water-holding capacity, and aggregate stability (Chia et al., 2022; Mbachu et al., 2021). These alterations can influence soil microbial community structure and activity, subsequently affecting soil nutrient cycling (Chen et al., 2020). Nitrogen, a vital element in soil biogeochemical processes, exists in various forms, including organic, inorganic, dissolved, ammonium, and nitrate. Soil nitrogen transformations are primarily driven by microorganisms and encompass processes such as nitrogen fixation, organic nitrogen degradation and synthesis, nitrification, denitrification, anammox, and nitrate reduction (Tu et al., 2018). The accumulation of microplastics may lead to shifts in nitrogen forms, potentially exacerbating serious environmental issues. These transformations can impact nitrogen availability, affecting soil fertility, agricultural pollution, and greenhouse gas emissions (Chuan et al., 2020; Greenfield et al., 2022; Yan et al., 2023). Numerous studies have examined the effects of microplastics on soil organic nitrogen, NH_4_^+^-N, and NO_3_^−^-N (Xu et al., 2019). While microplastics have been reported to influence the levels of various nitrogen forms, the observed changes have shown considerable variability, with positive, adverse, and negligible effects all reported (Shen et al., 2022; Dan et al., 2025; Contin et al., 2025). Microplastics disrupt microbial communities, including key nitrogen-cycling bacteria (e.g., *Nitrosomonas*, *Rhizobium*), and alter enzyme activities critical for nitrification and denitrification (Tang et al., 2025; Liu et al., 2023; Wang W. et al., 2024; Wang F. et al., 2024). Microplastics influence nitrogen transformation by affecting functional genes and microbial enzyme activities, which are essential for processes like denitrification and ammonification (Zhou et al., 2024). High concentrations of polypropylene and rubber crumb microplastics significantly impaired soil nitrogen cycling by inhibiting nitrification (reducing *amoA/amoC* gene abundance) and altering denitrification pathways, leading to decreased ammonium-N and nitrate-N availability in the soil (Liu et al., 2023). Plastic additives in polyethylene microplastics hinder soil nitrogen degradation and microbial immobilization more than the microplastics themselves, disrupting nitrogen cycling (Zhou et al., 2025). Microplastics negatively impact soil nitrogen cycling by altering microbial community structure and function, leading to inhibited nitrogen transformation rates and reduced nitrogen bioavailability. These effects are driven by changes in the abundance of key functional genes and the promotion of nitrogen loss pathways like denitrification (Huang et al., 2023; Ma et al., 2023; Shi et al., 2022; Zhou et al., 2024).

Biochar is a stable form of carbon produced through pyrolysis and is widely recognized as a climate change mitigation strategy (Lehmann et al., 2021). Numerous studies have shown the positive effects of using biochar in agricultural soils. Biochar improves soil fertility, rehabilitates degraded soil, alters nutrient cycling, and enhances nutrient use efficiency. Specifically, biochar shows promise in enhancing soil nitrogen retention and cycling (Lehmann et al., 2011; Liao et al., 2022). Its high surface area and porous structure enable it to absorb and retain ammonium (NH_4_^+^-N), reducing nitrogen leaching losses (Aralappanavar et al., 2024; Cao et al., 2017; Haider et al., 2017). Additionally, biochar provides a microhabitat for beneficial microbial communities, particularly those involved in nitrogen fixation and nitrification, while physically protecting soil organic matter from rapid decomposition (Lehmann et al., 2011; Zhu et al., 2017). These properties collectively promote nitrogen availability for plants and long-term soil fertility.

The recognition of individual and contrast effects of biochar and microplastics are growing, yet whether biochar’s nitrogen-stabilizing effects can offset the adverse impacts of microplastics, particularly across different polyethylene particle sizes remains untested. Besides, few studies have explored how biochar modulates microplastic-induced disruptions to microbial communities. Therefore, this study addressed the gaps by examining how biochar interacted with polyethylene-microplastics of varying particle sizes to influence soil nitrogen retention and microbial functionality. The primary objectives were: (1) to investigate the effects of different particle sizes of microplastics on soil nitrogen cycling; (2) to explore how biochar affect soil nitrogen cycling in microplastic-contaminated soil, and (3) to study the potential mechanism by which microplastic and biochar interact to affect soil nitrogen cycling. Our findings will inform strategies to mitigate microplastic pollution in agroecosystems while optimizing biochar applications for nitrogen management.

## 2 Material and method

### 2.1 Experimental material

Soil samples were collected from Zhongjiang County, Deyang City, Sichuan Province, to conduct this experiment. The soil collection site exhibits a temperate climate characterized by a mean annual temperature of 16.7 °C, annual precipitation of 883 mm, and a frost-free period of 286 days. The top 0–20 cm of uncultivated farmland soil was collected in a nylon bag. Plant roots and leaves were removed and the soil was air-dried at room temperature to constant weight. The soil properties were as follows: pH of 8.36, total organic carbon content of 7.15 g kg^−1^ and total nitrogen content of 1.12 g kg^−1^, soil available phosphorus content of 20.39 mg kg^−1^, soil available potassium content 80.56 mg kg^−1^ and no microplastics were detected. Three different sizes of polyethylene (PE) particles (1700 μm, 600 μm, and 6.5 μm) were selected for this experiment and were purchased from the Ruixiang Polymer Materials Trade Office, Zhangmutou, Dongguan, Guangdong, China. The corn straw biochar was purchased from Nanjing Qinfeng Zhongcheng Biomass New Materials Co., Ltd., China. Biochar was manually sieved through a 2-mm mesh and maintained at ambient temperature (~25 °C) with relative humidity < 50%. The physical and biochemical properties of corn straw biochar were determined as follows: carbon content 55%, hydrogen content 2.5%, oxygen content 41%, nitrogen content 1.1%, sulfur content 0.2%, pH 5.80 ± 0.01, redox potential 406.55 ± 1.70 mV and Zeta potential −38.40 ± 1.51 after mixing with the original soil.

### 2.2 Soil incubation test

The pretreated biochar was mixed with 150 g soil into a 250 mL culture bottle for the incubation experiment. The soil-microplastics-biochar mixture was pre-incubated at a constant temperature and humidity (25 ^o^ C, dark conditions) for 7 days to activate soil microbial activity and to create a suitable living environment for soil microorganisms. The concentration of microplastics was set at 0.5% *w/w*, selected based on (1) the typical application range of 0.05 to 60% (*w/w*) (Huang et al., 2024); and (2) a maximum polyethylene microplastic content of 0.5% *w/w* was found in agricultural fields in our previous study in Sichuan Province, China (Huang et al., 2025). The biochar concentration was determined at 1% *w/w*, reflecting its usage range in agricultural experiments from 1 to 5% *w/w* (Dari et al., 2016). Soils were treated with microplastics in three different sizes, i.e., 1700 μm, 600 μm, and 6.5 μm. The specific treatments were as follows: Control (without microplastic and biochar), B (only with biochar), 1700 (microplastic with particle size in 1700 μm), 600 (microplastic with particle size in 600 μm), 6.5 (microplastic with particle size in 6.5 μm), 1700 + B (microplastic with particle size in 1700 μm + biochar), 600 + B (microplastic with particle size in 600 μm + biochar), and 6.5 + B (microplastic with particle size in 6.5 μm + biochar) (see details in Table 1). All treatments were in triplicates. We monitored the water content daily to keep it at 60% of the soil water-holding capacity by adding deionized water. Soil samples were collected after being incubated for 0, 3, 7, 15, 30, and 45 days.

**Table 1:** Experimental treatments for the soil incubation test.

No.	Treatments in full name	Treatments in abbreviation	Biochar con. (% *w/w*)	Microplastic con. (% *w/w*)	Microplastic particle size (μm)
1	Control	Control	0	0	-
2	Biochar	B	1	0	-
3	Microplastic with particle size in 1700 μm	1700	0	0.5	1700
4	Microplastic with particle size in 600 μm	600	0	0.5	600
5	Microplastic with particle size in 6.5 μm	6.5	0	0.5	6.55
6	Microplastic with particle size in 1700 μm + biochar	1700 + B	1	0.5	1700
7	Microplastic with particle size in 600 μm + biochar	600 + B	1	0.5	600
8	Microplastic with particle size in 6.5 μm + biochar	6.5 + B	1	0.5	6.5

### 2.3 Soil nitrogen determination

The soil total nitrogen was measured with an elemental Analyzer (Vario EL Cube, Hanau, Germany). Briefly, the samples were digested in concentrated H_2_SO_4_ at elevated temperature, followed by distillation using alkali solution and titration with 0.01 mol L^−1^ H_2_SO_4_. Soil alkali-hydrolyzable nitrogen (AN) was quantified by the method of Roberts et al. (2011). Briefly, 5 g soil samples were distilled with 2 mol L^−1^ NaOH for 5 h and then with 10 mol L^−1^ NaOH for 7 min. Boric acid (40 g L^−1^) was used to absorb the liberated NH_3_ using the method of direct steam distillation. Soil alkali-hydrolyzable nitrogen content was quantified by conducting metric titration. Soil NH_4_^+^-N and NO_3_^−^-N concentrations were calculated using a continuous-flow analyzer (Skalar Analytical, Breda, Netherlands).

### 2.4 Microbiome profiling analysis

The composition of soil microbial communities was analyzed in collected soil samples at 0, 15 and 45 days. DNA was extracted from 0.5 g soil using OMEGA Soil DNA Kit (D5635-02, Omega Bio-Tek, Norcross, GA, USA) according to the manufacturer’s protocol. The 0.8% agarose gel electrophoresis (DYY-6C, Beijing Liuyi, Inc. Ltd., China) was used to resolve DNA fragments based on their molecular weight. Then the extracted DNA was quantified by Nanodrop (NC2000, Thermo Scientific, USA), followed by amplification of the 16S rRNA gene. The V3-V4 conservative region of bacteria of the 16S rRNA gene from the extracted DNA was sequenced using the PCR primers, i.e., 338F (5′-barcode + ACTCCTACG GGAGGCAGCA-3′) and 806R (5’-GGACTACHVGGGTWTCTAAT-3′). The PCR reaction used 5 μL of 5 × reaction buffer, 5 × high GC buffer (Q5^®^ High-Fidelity DNA Polymerase), 100 mM dNTP, 1 μL of 10 μM forward primer and 10 μM reverse primer, and 2 μL of DNA template, along with 8.75 μL ddH2O. The PCR amplification was done by initial DNA denaturation at 98 °C for 2 min, denaturation at 98 °C for 15 s, annealing at 59.5 °C for 30 s, extension at 72 °C for 30 s, final extension at 72 °C for 5 min, followed by 25–30 cycles held at 10 °C. The purified PCR products were adjusted to the same quantities and paired-end 2 × 250 base pair (bp) NovaSeq 6,000 SP Reagent Kit (500 cycles) for sequencing on an Illumina NovaSeq sequencing platform (Shanghai Personal Biotechnology Co., Ltd., China). The sequences were optimized and the quality was evaluated using the Quantitative Insights into Microbial Ecology (QIIME) pipeline.^1^ Based on the unique 10 bp barcodes, the reads were then assigned to each sample. The remaining sequences were clustered to operational taxonomic units (OTUs) with a threshold of 97% pairwise identity after removing barcode and primer sequences. The sequence reads then were annotated using BLAST searches against the Greengenes (Release 13.8, http://greengenes.secondgenome.com/, bacteria), where the most abundant bacterial OTUs from the soil samples, representing taxonomic identification, were checked using (QIIME).

### 2.5 Statistical analysis

The experimental data were subjected to rigorous statistical analysis to ensure validity and reliability. Soil sample distributions were tested for normality and homogeneity of variance before analysis, with results reported as mean ± standard error (*n* = 3). One-way ANOVA (*p* < 0.05) followed by Tukey’s *post hoc* test was performed using SPSS v27.0 to determine significant differences between treatment groups.

For microbial community analysis, bioinformatics processing was conducted in QIIME2 (2019.4), where ASV/OTU data were filtered (minimum mean abundance cut-off: 10–20) and classified taxonomically to genus/species levels when possible. Microbial diversity was characterized using *α*-diversity indices (Chao1, ACE, Shannon, Simpson, and Pielou’s evenness) and *β*-diversity metrics (PCoA and hierarchical clustering based on Bray-Curtis distance). Community structural differences were verified through PERMANOVA with weighted UniFrac distances. Microbial co-occurrence networks were constructed based on Spearman correlation analysis. Only correlations that were statistically significant (*p* < 0.05 after false discovery rate (FDR) correction) were retained for network construction to minimize false-positive associations. Furthermore, only interactions that were consistently observed across all three biological replicates within each treatment were included in the final network analysis to ensure robustness. The overall differences in microbial community composition among treatments were further validated using permutational multivariate analysis of variance (PERMANOVA, *p* < 0.05). Microbial composition patterns were visualized via heatmaps, while ecological interactions were examined using co-occurrence networks constructed in Cytoscape v3.10.1.

## 3 Results

### 3.1 The effect of microplastic and biochar on soil nitrogen variations

#### 3.1.1 Total nitrogen variation

**Figure 1:**
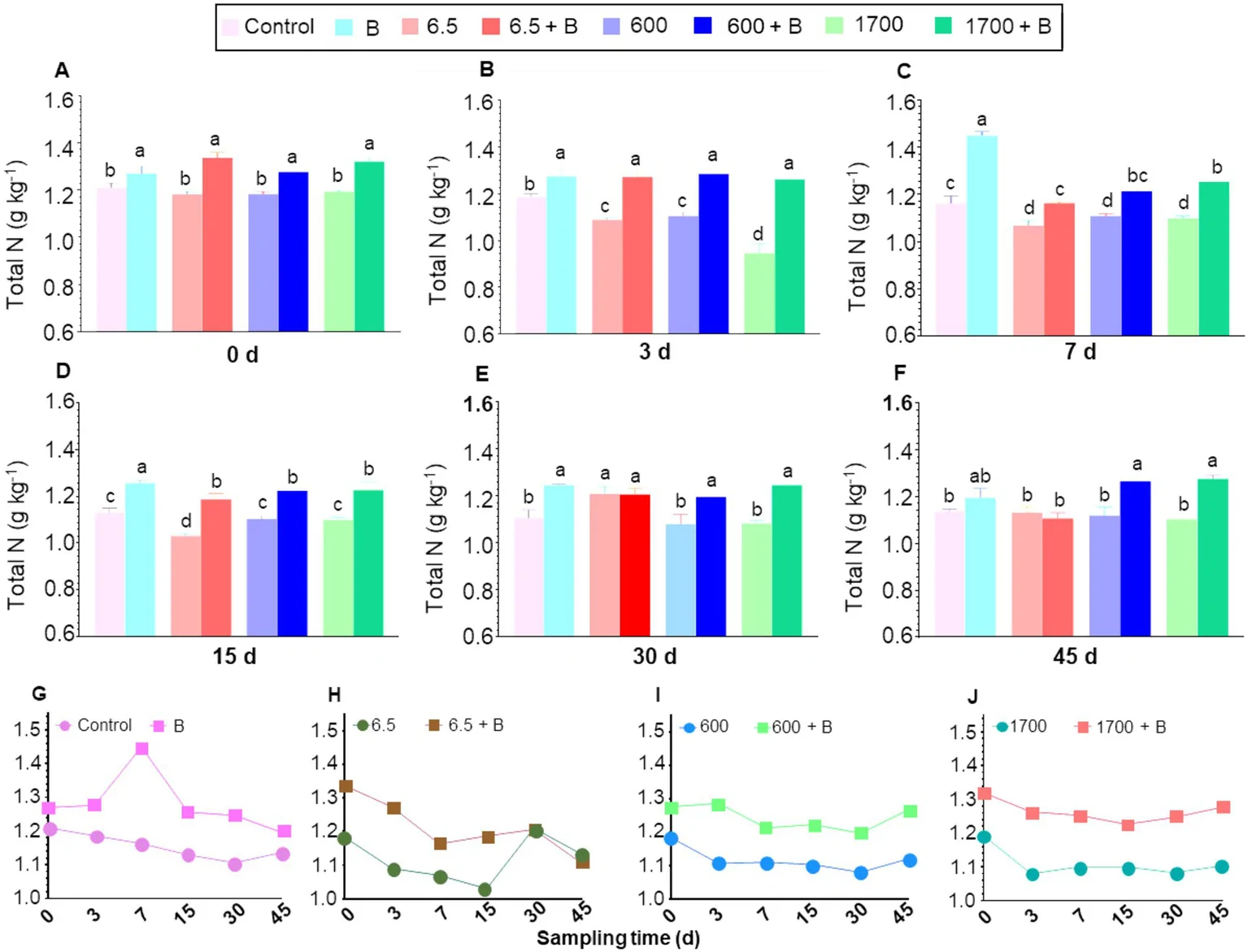
Total nitrogen contents in different treatments after 0, 3, 7, 15, 30, and 45 days of incubation with microplastics and biochar. **(A–F)** Total nitrogen contents at 0, 3, 7, 15, 30, and 45 days of incubation, respectively. **(G–J)** Changes in total nitrogen contents over the incubation period for Control and B treatments **(G)**, 6.5 and 6.5 + B treatments **(H)**, 600 and 600 + B treatments **(I)**, and 1700 and 1700 + B treatments **(J)**. Different lowercase letters indicate statistically significant differences among groups based on one-way ANOVA and HSD (*p* < 0.05). B–Biochar; 6.5–6.5 μm microplastics; 6.5 + B–6.5 μm microplastics + biochar; 600–600 μm microplastics; 600 + B–600 μm microplastics + biochar; 1700–1700 μm microplastics; 1700 + B– 1700 μm microplastics + biochar.

The total nitrogen content in the soil after 0, 3, 7, 15, 30, and 45 days of incubation was determined in different microplastic sizes and biochar treatments (Figures 1A–F). On initial days (3, 7, and 15) days, microplastic treatments exhibited a decrease in total nitrogen as compared to the control (Figures 1D–F). On 30 and 45 days, a non-significant difference between the control and microplastic treatments was observed (Figures 1E,F). Although significant differences among individual microplastic-size treatments were observed during the early incubation period (3–15 days), these differences diminished over time. By days 30 and 45, total nitrogen concentrations among the microplastic-only treatments were comparable indicating that microplastic particle size did not exert a consistent effect on total nitrogen throughout the incubation period. We observed that the microplastic mixed with biochar improved the total nitrogen contents compared to the control (*p* < 0.05). On 3, 7, and 15 days after incubation, adding biochar along with microplastic significantly improved the total nitrogen in all sizes of microplastic treatments (*p* < 0.05). Notably, in 30 and 45 days, biochar application significantly impacted 600 and 1700 μm microplastic treatments but had a non-significant (*p* < 0.05) impact on 6.5 μm microplastic treatment. In all treatments, the highest total nitrogen content was observed in the biochar treatment (1.45 g kg^−1^) at 7 days of incubation (Figure 1C), and the lowest total nitrogen content was observed in the 1700 μm microplastic treatment after 3 days of incubation (Figure 1B). With the increase of time, the total nitrogen in all treatments tended to get stable. Only a significant (*p* < 0.05) increase in biochar (7 days after incubation) and 6.5 μm microplastics (15 days after incubation) treatment was exhibited.

#### 3.1.2 NO_3_^−^-N variation

**Figure 2:**
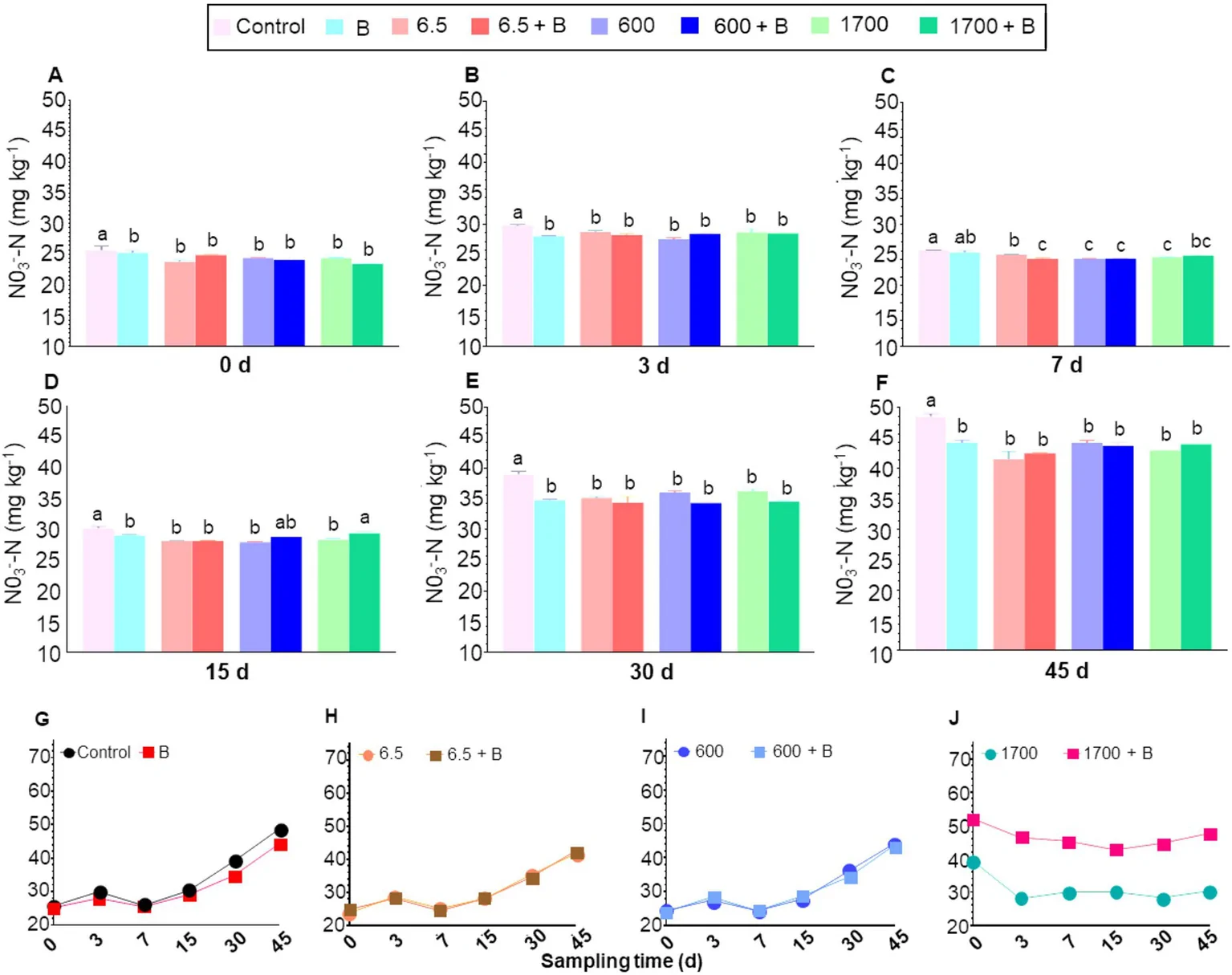
NO_3_^−^-N contents in different treatments after 0, 3, 7, 15, 30, and 45 days of incubation with microplastics and biochar. **(A–F)** NO_3_^−^-N contents at 0, 3, 7, 15, 30, and 45 days of incubation, respectively. **(G–J)** Changes in NO_3_^−^-N contents over the incubation period for Control and B treatments **(G)**, 6.5 and 6.5 + B treatments **(H)**, 600 and 600 + B treatments **(I)**, and 1700 and 1700 + B treatments **(J)**. Different lowercase letters indicate statistically significant differences among groups based on one-way ANOVA and HSD (*p* < 0.05). B–Biochar; 6.5–6.5 μm microplastics; 6.5 + B–6.5 μm microplastics + biochar; 600–600 μm microplastics; 600 + B–600 μm microplastics + biochar; 1700–1700 μm microplastics; 1700 + B–1700 μm microplastics + biochar.

The changes in soil nitrate (NO_3_^−^-N) content were measured at six sampling time (days 0, 3, 7, 15, 30, and 45) during the incubation experiment. All treatments at 0, 3, 7, and 15 days exhibited non-significant differences (Figures 2A–C) with an increase trend in NO_3_^−^-N contents. However, after 30 days, an increase in NO_3_^−^-N contents were observed in all treatments (Figures 2E,F). A non-significant impact of biochar in different sizes of microplastic-polluted soil on NO_3_^−^-N contents was observed (Figures 2H–J). Only a tiny increase was observed in NO_3_^−^-N contents in biochar treatment as compared to the control at 30 and 45 days of incubation (Figure 2G). The lowest NO_3_^−^-N contents in all treatments were observed on day 7, and the highest contents were observed on 45 days of incubation.

#### 3.1.3 NH_4_^+^-N

**Figure 3:**
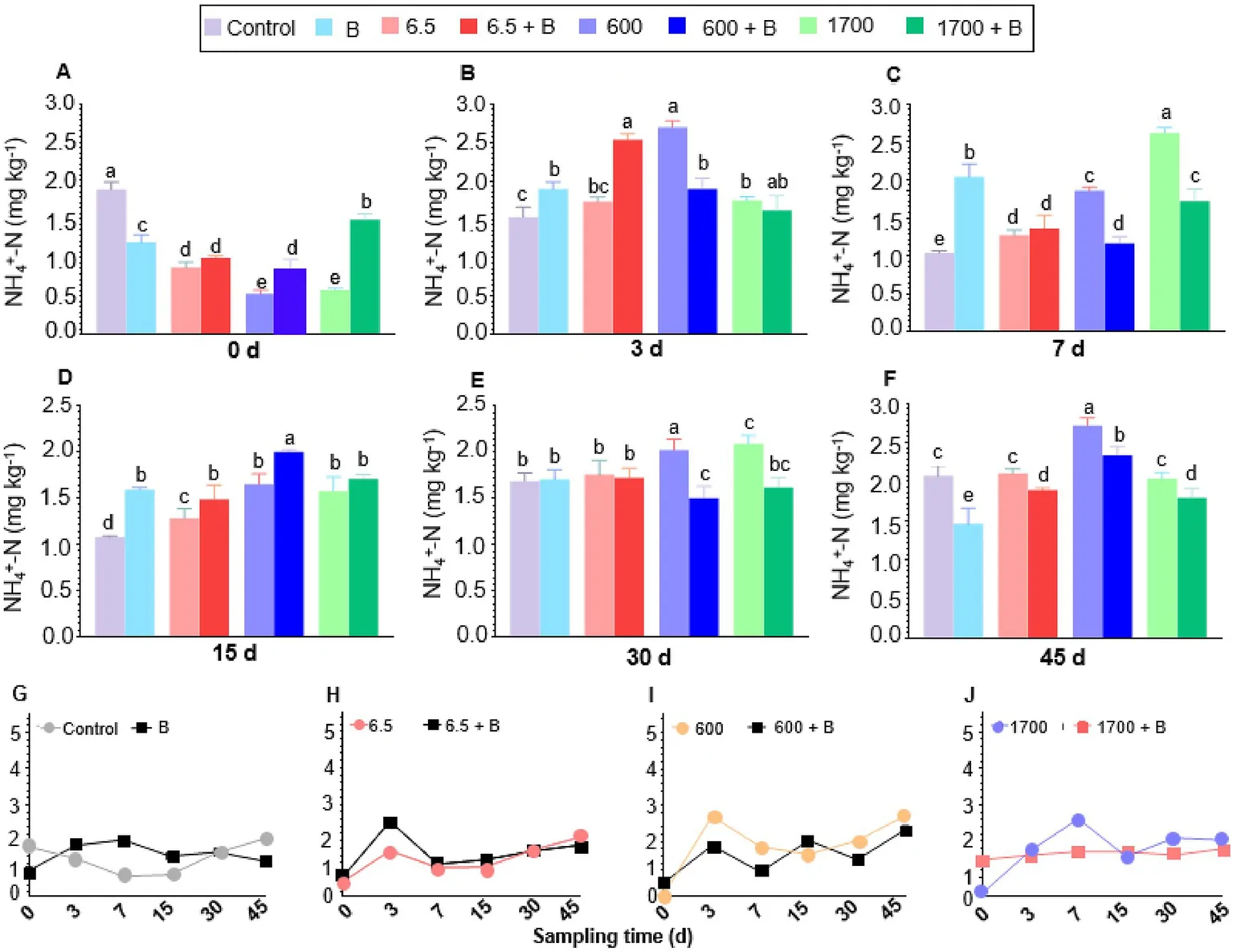
NH_4_^+^-N contents in different treatments after 3, 7, 15, 30, and 45 days of incubation with microplastics and biochar. **(A–F)** NH_4_^+^-N contents at 0, 3, 7, 15, 30, and 45 days of incubation, respectively. **(G–J)** Changes in NH_4_^+^-N contents over the incubation period for Control and B treatments **(G)**, 6.5 and 6.5 + B treatments **(H)**, 600 and 600 + B treatments **(I)**, and 1700 and 1700 + B treatments **(J)**. Different lowercase letters indicate statistically significant differences among groups based on one-way ANOVA and HSD (*p* < 0.05). B–Biochar; 6.5–6.5 μm microplastics; 6.5 + B–6.5 μm microplastics + biochar; 600–600 μm microplastics; 600 + B–600 μm microplastics + biochar; 1700–1700 μm microplastics; 1700 + B–1700 μm microplastics + biochar.

The fluctuation in NH_4_^+^-N levels over time was observed under microplastics and biochar treatments. The influence of biochar onNH_4_^+^-N level varied with incubation time and microplastic particle size. Biochar significantly increased NH_4_^+^-N under the 6.5 μm and 600 μm treatments during the early incubation period (3–15 days), whereas treatment differences became less pronounced during the later incubation period. The highest NH_4_^+^-N level was observed at 45 days of incubation when 600 μm microplastic was applied (Figure 3).

#### 3.1.4 Alkaline-hydrolyzable nitrogen

**Figure 4:**
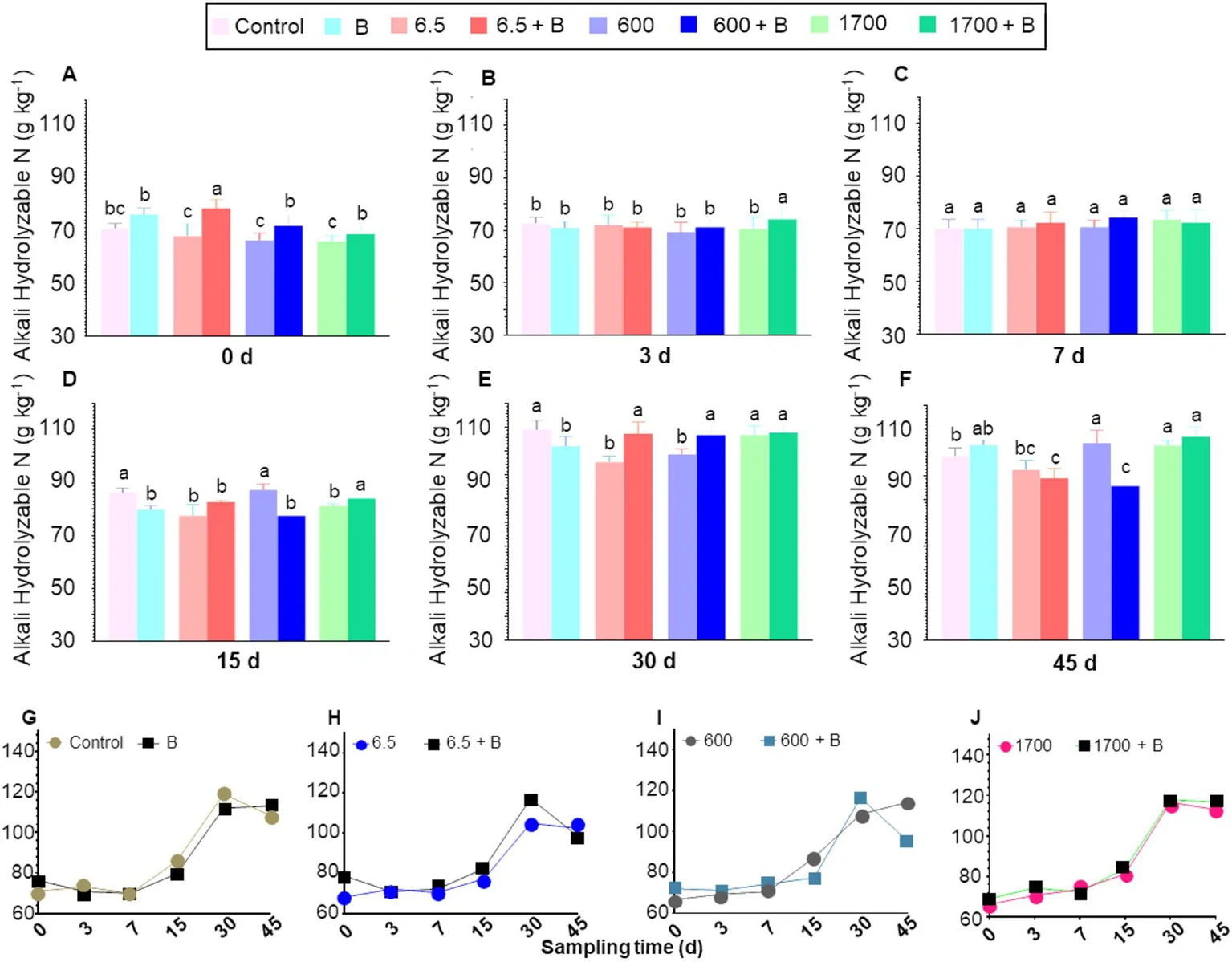
Alkali-hydrolyzable nitrogen contents in different treatments after 0, 3, 7, 15, 30, and 45 days of incubation with microplastics and biochar. **(A–F)** Alkali-hydrolyzable nitrogen contents at 0, 3, 7, 15, 30, and 45 days of incubation, respectively. **(G–J)** Changes in alkali-hydrolyzable nitrogen contents over the incubation period for Control and B treatments **(G)**, 6.5 and 6.5 + B treatments **(H)**, 600 and 600 + B treatments **(I)**, and 1700 and 1700 + B treatments **(J)**. Different lowercase letters indicate statistically significant differences among groups based on one-way ANOVA and HSD (*p* < 0.05). B–Biochar; 6.5–6.5 μm microplastics; 6.5 + B–6.5 μm microplastics + biochar; 600–600 μm microplastics; 600 + B–600 μm microplastics + biochar; 1700–1700 μm microplastics; 1700 + B–1700 μm microplastics + biochar.

Adding biochar did not increase alkaline-hydrolyzable nitrogen content compared to the control (Figure 4). The degree of inhibition of soil alkaline-hydrolyzable nitrogen by different microplastic sizes varied slightly. Smaller particle sizes (6.5 μm and 600 μm) significantly decreased alkaline-hydrolyzable nitrogen compared to the control (*p* < 0.05) on all days, while the larger particle size (1700 μm) increased the content until after 45 days of incubation. When smaller-sized microplastic was mixed with biochar, alkaline-hydrolyzable nitrogen levels were slightly increased. When biochar was combined with a larger particle size (1700 μm), no difference was observed in hydrolyzable nitrogen levels between microplastics and microplastics with biochar treatments. The highest levels occurred when biochar was mixed with 600 μm particle size microplastic at 45 days of incubation, and the lowest was observed in 600 μm treatment on the 3rd day of incubation.

### 3.2 Microplastic size and biochar effect on soil microbial community

**Figure 5:**
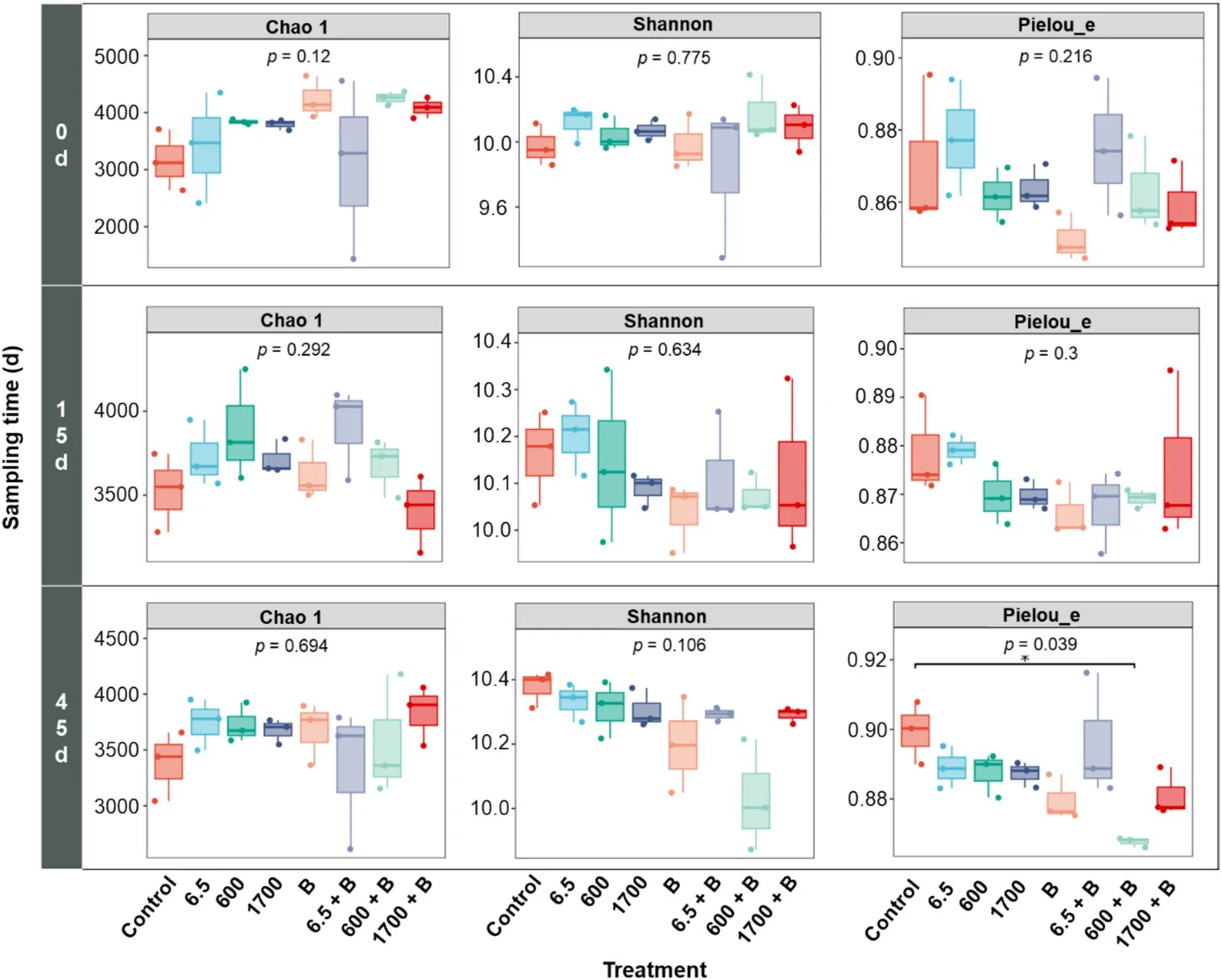
Box-plot showing bacterial *α*-diversity indices (Chao1, Shannon, and Pielou evenness) in soil samples treated with microplastics and biochar at 0, 15, and 45 days. Significant differences among groups at *p* < 0.05 (Dunn’s post test). B–Biochar; 6.5–6.5 μm microplastics; 6.5 + B–6.5 μm microplastics+biochar; 600–600 μm microplastics; 600 + B–600 μm microplastics+biochar; 1700–1700 μm microplastics; 1700 + B–1700 μm microplastics+biochar.

Alpha species diversity within the sample was measured using (Iqbal et al., 2024), Shannon, and Simpson indices. The Chao1 and Simpson indices generally increased across all treatments, indicating a rise in species richness and evenness, respectively. However, the Shannon index, which considers richness and evenness, exhibited a more complex pattern. While some treatments showed an increase in the Shannon index over time, others remained relatively stable or decreased. The 1700 μm microplastics with biochar treatment resulted in distinct microbial community profiles compared to the control. This was particularly evident at day 45, where the 1700 μm microplastics with biochar treatment significantly increased the Chao1 index, indicating higher microbial richness. However, the Shannon index for this treatment remained relatively low, suggesting that a proportional increase in evenness did not accompany the increased richness. Across all treatments, microbial community structure underwent significant shifts over time (Figure 5).

**Figure 6:**
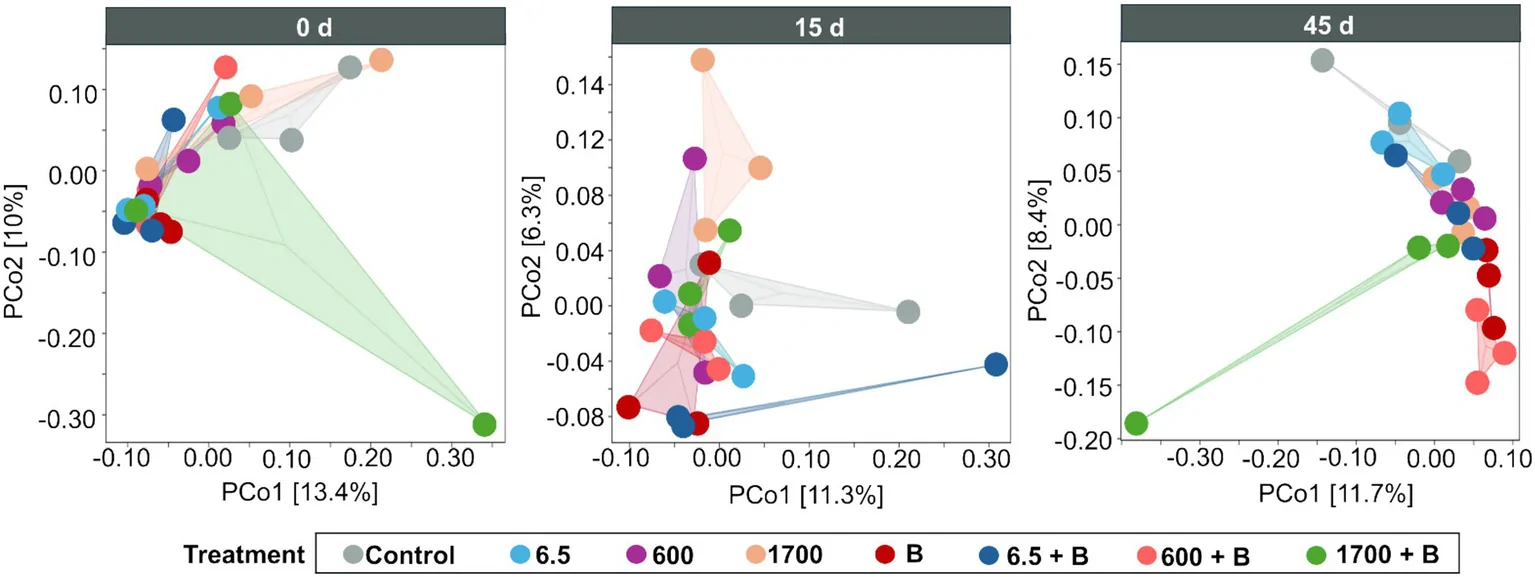
Microbial principal coordinates analysis (PCoA) of *β* diversity at the species level in soil samples treated with microplastics and biochar at 0, 15 and 45 days. B–Biochar; 6.5–6.5 μm microplastics; 6.5 + B–6.5 μm microplastics + biochar; 600–600 μm microplastics; 600 + B–600 μm microplastics + biochar; 1700–1700 μm microplastics; 1700 + B–1700 μm microplastics + biochar.

The β diversity was visualized using principal coordinates analysis (PCoA) across day 0, 15, and 45 (Figure 6). At day 0, prior to treatment application, all samples clustered closely together. By day 15, a clear separation emerged along PCo1. The control group maintained a central position. The treatments formed two distinct clusters: one comprising the 6.5 μm, 600 μm, and 1700 μm microplastics groups, which overlapped slightly with the control, and a second, more separated cluster containing the biochar-amended groups (Biochar, 6.5 μm microplastics + biochar, 600 μm microplastics + biochar, 1700 μm microplastics + biochar). At day 45, the separation between treatments became more pronounced. The control community shifted from its original position. The non-biochar-amended treatments (6.5 μm, 600 μm, and 1700 μm microplastics) formed a cluster that was distinct from both the control and the biochar-amended groups. Notably, the biochar-amended groups (Biochar, 6.5 μm microplastics + biochar, 600 μm microplastics + biochar, 1700 μm microplastics + biochar) formed a tight, well-separated cluster.

**Figure 7:**
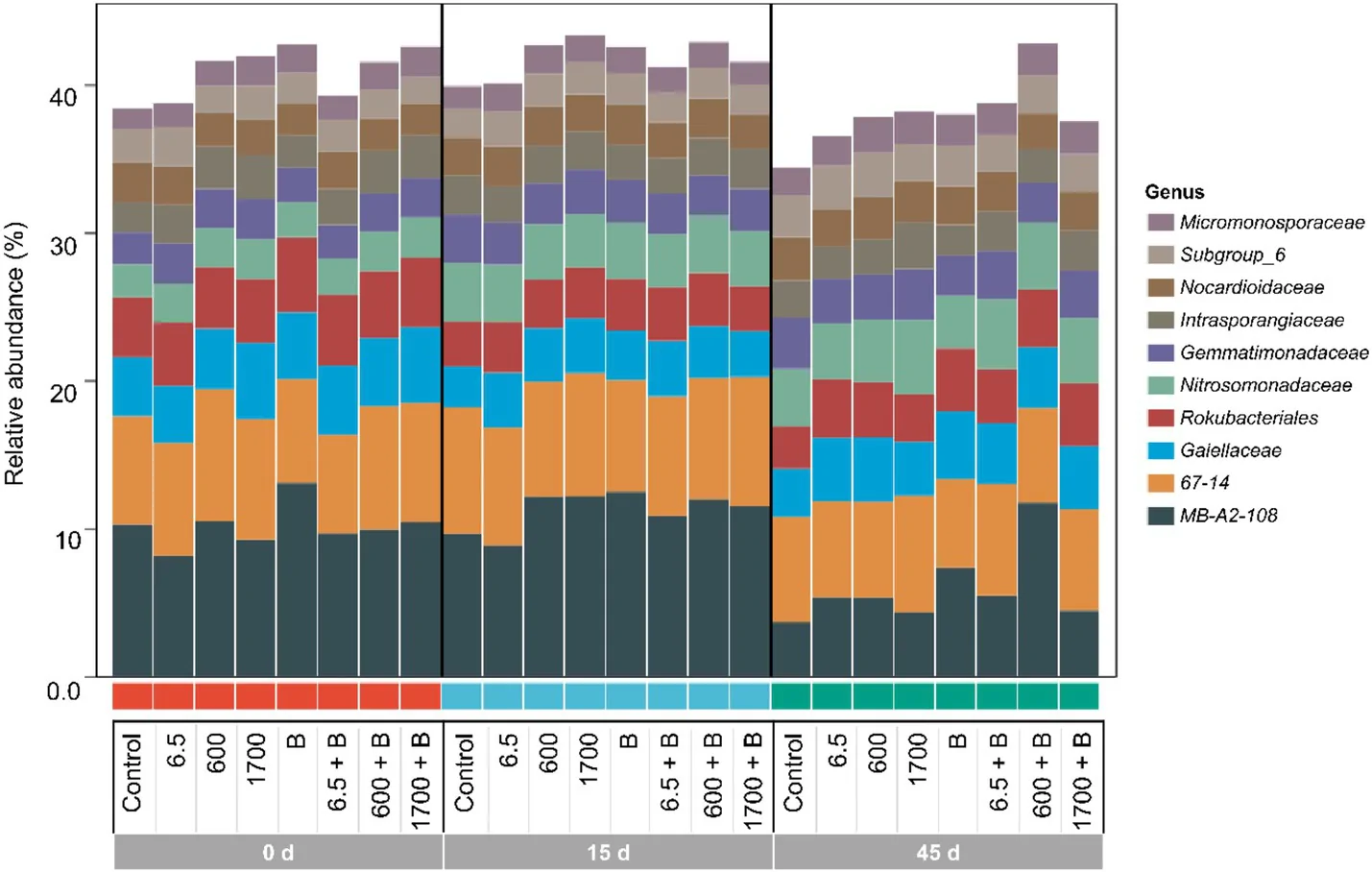
Relative abundance of the dominant bacterial genera across different treatments and incubation times: B–Biochar; 6.5–6.5 μm microplastics; 6.5 + B–6.5 μm microplastics + biochar; 600–600 μm microplastics; 600 + B–600 μm microplastics + biochar; 1700–1700 μm microplastics; 1700 + B–1700 μm microplastics + biochar.

The relative abundance of the dominant bacterial genera varied among treatments and incubation times (Figure 7). Biochar amendment influenced the composition of the dominant bacterial community, with noticeable shifts observed after 15 and 45 days of incubation. Differences in the relative abundance of several dominant genera were evident among the three microplastic particle-size treatments, indicating that particle size affected the overall bacterial community composition. Temporal changes in community structure were also observed throughout the incubation period, with some dominant genera increasing in relative abundance while others declined. Overall, the 1700 μm microplastic treatment combined with biochar exhibited the most distinct shift in the dominant bacterial community after 45 days, suggesting that larger polyethylene microplastics, together with biochar amendment, altered the composition of the dominant bacterial community under the experimental conditions of this study.

**Figure 8:**
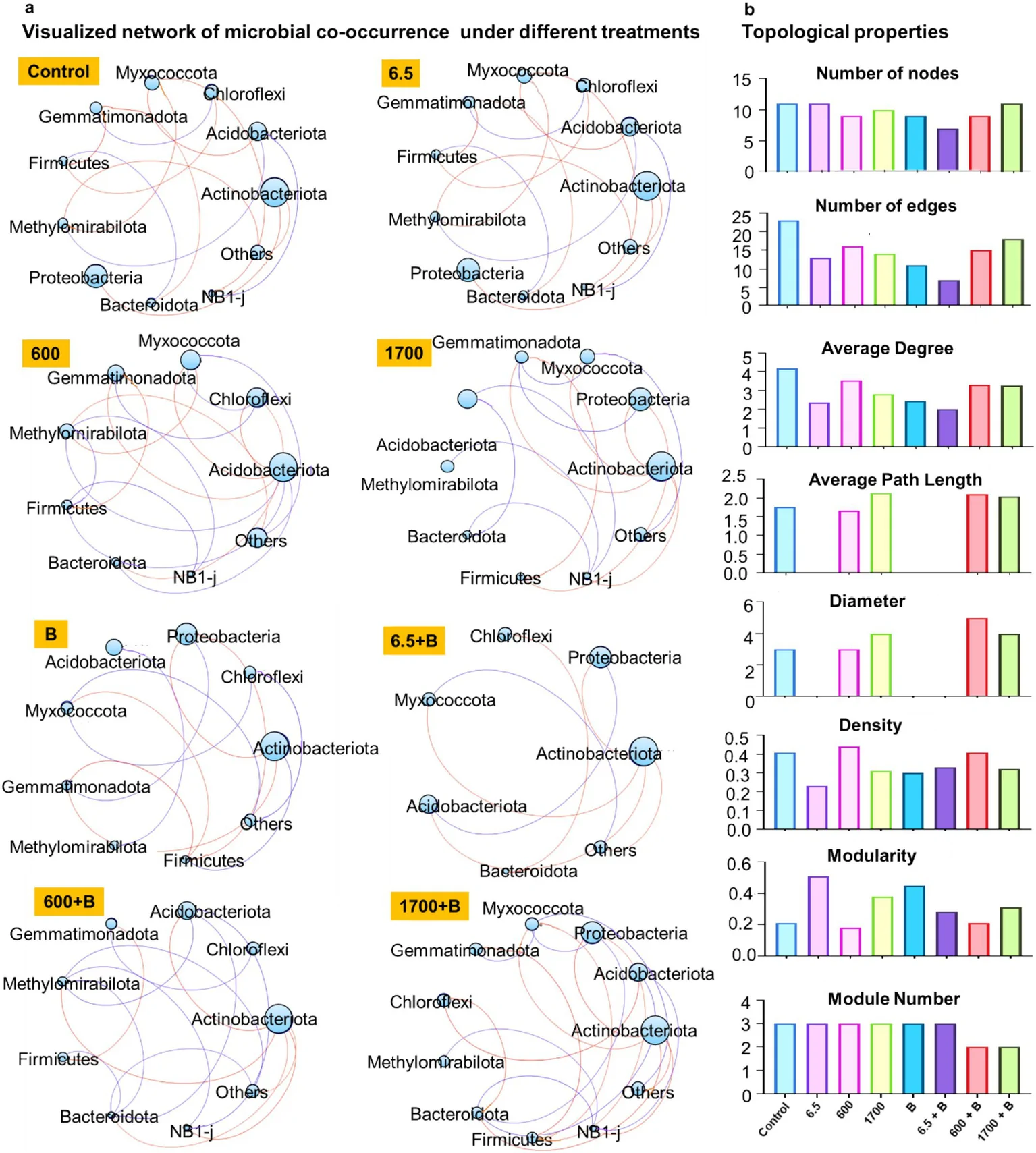
Visualized networks of microbial co-occurrence patterns **(a)** and the topological properties **(b)** in in soil samples treated with microplastics and biochar at 0, 15 and 45 days. B–Biochar; 6.5–6.5 μm microplastics; 6.5 + B–6.5 μm microplastics + biochar; 600–600 μm microplastics; 600 + B–600 μm microplastics + biochar; 1700–1700 μm microplastics; 1700 + B–1700 μm microplastics + biochar.

Bacterial co-occurrence network analysis was conducted at the OTU level to further explore the complexity of the microbial community relationships among the different microplastics and biochar treatments. The bacterial co-occurrence networks showed distinct patterns among treatments (Figure 8A). The control and biochar amended networks were the most complex, characterized by a dense web of interactions and the presence of numerous highly connected hub genera. The 6.5, 600 and 1700 μm microplastic treatments each resulted in a dramatic reduction in network complexity and connectivity. The biochar addition partially mitigated these effects. The combined treatments (Biochar, 6.5 μm microplastics + biochar, 600 μm microplastics + biochar, 1700 μm microplastics + biochar) displayed more complex network than their counterparts without biochar. With increasing microplastic concentration, the network size represented by the number of nodes and modularity showed a general declining trend, whereas network connectivity, indicated by the number of edges, average degree, and density, displayed an overall increase. In contrast, parameters related to network architecture and efficiency, including average path length, diameter, and module number, fluctuated across treatments, suggesting differential reorganization of microbial interactions under stress (Figure 8B). Notably, under high microplastic stress 1700 μm the bacterial network exhibited reduced size and complexity, yet displayed enhanced connectivity and integration, as reflected by higher edge numbers and density. The most compact and highly connected network structure was observed under the 600 μm microplastic treatment, which also coincided with the highest network efficiency in terms of substance circulation, energy flow, and information exchange among bacterial taxa. Furthermore, the addition of biochar modulated these effects, often increasing network complexity and stability particularly in combination with lower microplastic levels as evidenced by higher modularity and node counts in treatments such as 6.5 + B (6.5 μm microplastics + biochar) and biochar alone.

### 3.3 Correlation among soil nitrogen and bacterial variables

**Figure 9:**
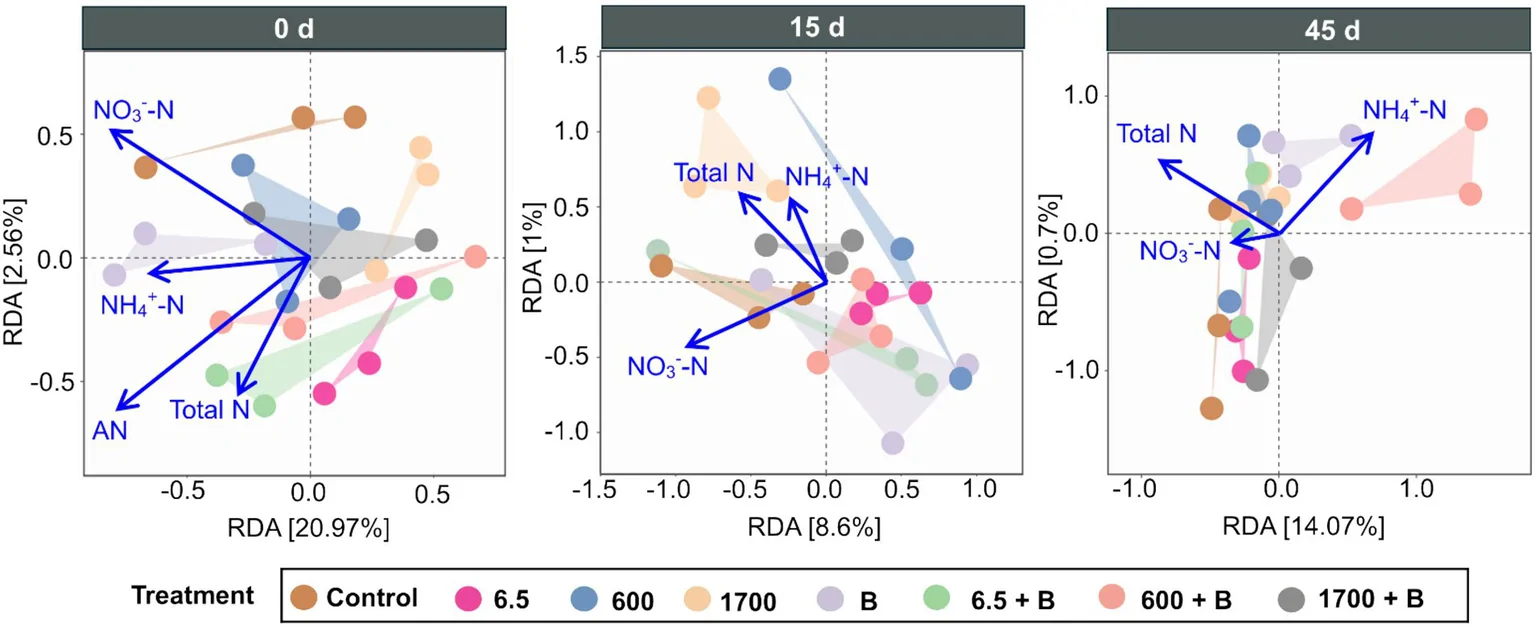
Redundancy analysis (RDA) illustrates the dynamic relationships between soil nitrogen variables and microbial community structure, revealing a strengthening treatment effect over time. Redundancy analysis (RDA) for soil nitrogen and bacterial community structure in soil samples treated with microplastics and biochar at 0, 15, and 45 days. The length and direction of the arrow represent the degree of influence of soil nitrogen on the structure of bacterial community, and the positive and negative correlation between them. B–Biochar; 6.5–6.5 μm microplastics; 6.5 + B–6.5 μm microplastics + biochar; 600–600 μm microplastics; 600 + B–600 μm microplastics + biochar; 1700–1700 μm microplastics; 1700 + B–1700 μm microplastics + biochar.

To explore the correlation between soil nitrogen and soil bacterial community, redundancy analysis (RDA) was performed using Canoco v5.0 software (Figure 9). At day 0, the first two RDA axes explained a minimal amount of variance (2.56 and 1.0%, respectively). The environmental factor vectors were short, and all treatment groups clustered tightly together. By day 15, a slight separation of treatments began to emerge along RDA1 (20.97% explained variance). The NO_3_^−^-N vector pointed strongly to the right, opposing the NH_4_^+^-N and Total N vectors which pointed to the left. The biochar-amended treatments (Biochar, 6.5 μm microplastics + biochar, 600 μm microplastics + biochar, 1700 μm microplastics + biochar) had a positive association with NO_3_^−^-N at this stage. The non-amended treatments (Control, 6.5, 600, 1700 μm microplastics) remained more centrally located or slightly associated with the NH_4_^+^-N and total nitrogen axis. At day 45, the model’s explanatory power was greatest, with RDA1 explaining 14.07% of the variance. The biochar-amended groups of 6.5 μm microplastics + biochar formed a distinct cluster strongly associated with high NH_4_^+^-N availability. In contrast, the non-amended groups, particularly the control and microplastics, were positioned in the opposite direction, showing a stronger association with NH_4_^+^-N and total N. To summarize, the RDA revealed that the relationship between microbial community structure and soil nitrogen dynamics became progressively stronger over 45 days.

## 4 Discussion

The present study demonstrated that biochar amendment played a regulating role in soil nitrogen dynamics under microplastic stress, with its effects differing among nitrogen forms and microplastic particle sizes. Overall, biochar increased soil total nitrogen (TN) content across all microplastic treatments, while its effects on nitrate (NO₃^−^–N), ammonium (NH₄^+^–N), and alkali-hydrolyzable nitrogen were comparatively limited and dependent on microplastic particle size and incubation time. These findings highlight that biochar functions primarily as a stabilizer of bulk soil nitrogen rather than uniformly enhancing all plant-available nitrogen forms in microplastic-polluted soils.

The findings from our study are broadly consistent with previous reports showing that polyethylene MP can influence soil nitrogen cycling, although the underlying mechanisms may vary among experimental systems (Iqbal et al., 2024; Liu et al., 2023; Xiang et al., 2023). For example (Yu et al., 2025) reported that polyethylene MPs reduced gross nitrification rates and decreased AOA *amoA* gene abundance in rice paddy soils. Because functional genes and nitrification rates were not measured in the present study, direct comparisons with those mechanisms cannot be made. Nevertheless, we observed that microplastic particle size was associated with distinct shifts in bacterial community composition, with the smallest microplastics (6.5 μm) showing a higher relative abundance of *Vicinamibacteraceae*, whereas the largest microplastics (1700 μm) were associated with increased relative abundances of *Sphingomonas* and *Lysobacter*. These community shifts may represent one possible explanation for the different nitrogen responses observed among particle-size treatments (Yu et al., 2025), but this hypothesis requires verification using functional gene analyses and direct measurements of nitrogen transformation processes.

Similarly, Lin et al. (2025) reported that increased N₂O emissions and changes in *amoA*-containing microorganisms in polyethylene microplastic-treated paddy soils. Although our results also indicate that biochar altered bacterial community composition under microplastic stress, our study did not quantify N₂O or nitrogen-cycling functional gene. Therefore, we cannot conclude that the microbial community changes observed here resulted in altered nitrification, denitrification, or N_2_O production. Instead, these previous studies provide useful context suggesting potential mechanisms that may contribute to the nitrogen responses observed in our experiment. Collectively, these findings indicate that the influence of microplastics on soil nitrogen cycling is likely to depend on multiple interacting factors, including particle size, soil properties, and microbial community responses. Additional studies integrating microbial functional genes, process-level nitrogen transformation, and greenhouse gas measurements would help clarify these mechanisms.

Biochar amendment consistently increased TN content in microplastic-polluted soils regardless of microplastic particle size, particularly during the early and mid-incubation stages. This response aligns with previous studies demonstrating that biochar enhances soil nitrogen retention (Cao et al., 2017; Haider et al., 2017; Kammann et al., 2015) by increasing adsorption sites, improving aggregation, and physically protecting organic nitrogen from rapid mineralization (Lehmann et al., 2003; Joseph et al., 2010; Zhang et al., 2016). The observed TN increase suggests that biochar mitigates microplastic-induced nitrogen depletion by reducing nitrogen losses rather than stimulating nitrogen transformation processes.

Although microplastic treatments alone initially reduced TN, especially during the early incubation period, these effects weakened over time, and TN levels stabilized by days 30 and 45. This temporal pattern indicates that microplastic-induced nitrogen disturbance may be transient under controlled incubation conditions, possibly due to microbial adaptation or redistribution of nitrogen into more stable pools. Importantly, the consistent TN enhancement across all microplastic sizes suggests that biochar’s nitrogen-retention capacity is largely independent of microplastic particle size, supporting its potential use as a general amendment for maintaining soil nitrogen stocks in microplastic-contaminated systems.

In contrast to TN, biochar exhibited a size-dependent influence on soil NO_3_^−^–N. Biochar amendment did not significantly affect NO_3_^−^–N concentrations in soils contaminated with smaller microplastic particles (6.5 and 600 μm), whereas a clear increase in NO_3_^−^–N was observed in soils containing larger microplastics (1700 μm), particularly at later incubation stages. This result suggests that biochar does not universally enhance nitrification or nitrate retention under microplastic stress.

The lack of biochar effect on NO_3_^−^–N in smaller particle size treatments may reflect stronger microbial or physicochemical interference induced by finer microplastics, which have higher surface area and reactivity. Smaller microplastics may disrupt nitrifying microbial niches or adsorb inorganic nitrogen more effectively, thereby constraining nitrate accumulation even in the presence of biochar. In contrast, larger microplastics exert a weaker disturbance on soil structure and microbial functioning, allowing biochar-associated improvements in aeration, microbial habitat, or substrate availability to support nitrate formation and accumulation. Similar size-dependent responses have been reported in previous studies where microplastic particle size influenced nitrification efficiency and nitrate availability (De Souza Machado et al., 2019; Zhou et al., 2025).

Biochar amendment tended to decrease NH_4_^+^–N concentrations in microplastic-polluted soils, although the magnitude of this effect was minor and inconsistent across treatments. This finding indicates that biochar did not function primarily as an ammonium retainer under the experimental conditions, our finding is consistent with (Feng et al., 2021), who demonstrated that biochar and microplastic interactions can influence soil nitrogen dynamics. The relatively weak response observed in the present study may therefore reflect the combined effects of biochar properties, microplastic characteristics, and soil conditions. Instead, the slight reduction in NH_4_^+^–N may reflect enhanced microbial utilization or transformation of ammonium rather than direct physicochemical immobilization. Previous studies have shown that biochar can adsorb NH_4_^+^–N through cation exchange mechanisms (Aralappanavar et al., 2024); however, such effects are strongly soil-dependent and may be offset by biochar-induced stimulation of microbial activity. In microplastic-polluted soils, where microbial communities are already altered, biochar may facilitate ammonium oxidation or microbial assimilation rather than retention. The limited magnitude of NH_4_^+^–N change observed in this study suggests that ammonium dynamics are governed more by microplastic-mediated microbial processes than by biochar amendment alone.

Biochar exerted only a minor effect on alkali-hydrolyzable nitrogen content across all microplastic particle sizes and incubation periods. This indicates that biochar did not substantially alter the potentially mineralizable organic nitrogen pool under microplastic stress. Alkali-hydrolyzable nitrogen is often associated with relatively stable organic nitrogen fractions, and its limited response suggests that neither microplastics nor biochar strongly influenced the transformation of these pools within the experimental timeframe. The weak response of alkali-hydrolyzable nitrogen is consistent with previous findings showing that biochar primarily affects labile nitrogen forms and nitrogen retention rather than accelerating the decomposition of stable organic nitrogen fractions (Major et al., 2010; Schimel and Bennett, 2004). Moreover, microplastic-induced changes in soil structure and microbial composition may preferentially impact inorganic nitrogen pathways rather than slowly cycling organic nitrogen pools.

Changes in soil nitrogen forms were accompanied by shifts in bacterial community composition, indicating that microbial community responses were associated with nitrogen dynamics under combined microplastic and biochar treatments. The present study showed that biochar amendment influenced the composition of the dominant bacterial community in soils exposed to polyethylene microplastics which is consistent with the findings of Zhang et al. (2018), who reported that biochar amendment can significantly alter soil microbial community composition. Similarly, Dominchin et al. (2021) demonstrated that biochar amendment influenced soil microbial community structure and microbial activity. Massaccesi et al. (2025) further reported that HDPE microplastic exposure affected soil microbial community composition, highlighting the sensitivity of soil microbial communities to microplastic stress. More directly, Ran et al. (2023) showed that biochar amendment altered bacterial community composition and predicted microbial functions in microplastic-contaminated soil. Collectively, these findings support the potential role of biochar in modifying soil microbial communities under microplastic stress, although the magnitude and direction of microbial responses may depend on microplastic characteristics, biochar properties, and experimental conditions. This suggests that biochar may play an important role in reshaping soil bacterial communities, even under microplastic-induced environmental stress. Differences in dominant bacterial community composition were observed among the three microplastic particle-size treatments, with the most distinct community shift occurring in the 1700 μm microplastic treatment after 45 days of incubation. These findings suggest that microplastic particle size, together with biochar amendment, may influence the structure of dominant bacterial communities under the experimental conditions of this study. However, because this study was based on 16S rRNA amplicon sequencing, these observations describe changes in community composition and should not be interpreted as evidence of direct functional or mechanistic effects on soil nitrogen transformations. Further studies integrating functional gene analyses and metagenomic approaches are needed to clarify the microbial mechanisms underlying these responses. Functional genes, microbial activities, and nitrogen transformation rates were not measured; therefore, causal relationships cannot be established from the present dataset.

These findings indicate that biochar has the potential to mitigate certain microplastic-induced changes in soil nitrogen dynamics under the controlled conditions evaluated in this study. However, because only one soil type, one biochar source, one polyethylene microplastic concentration, and one biochar application rate were examined, caution should be exercised when extrapolating these results to other agricultural soils or environmental conditions. Further field-based studies across diverse soil types and management systems are needed to evaluate the general applicability of these observations.

## 5 Conclusion

This study demonstrated that polyethylene microplastic particle size influenced soil nitrogen dynamics and microbial community composition under controlled incubation conditions. Biochar amendment consistently increased soil total nitrogen and modified microbial community structure, although its effects on nitrate, ammonium, and alkali-hydrolyzable nitrogen varied depending on microplastic particle size and incubation time. These findings suggest that corn straw biochar may improve soil nitrogen availability in polyethylene microplastic-contaminated soil under the specific experimental conditions investigated. However, because this study was conducted using a single soil type, one biochar feedstock, one microplastic polymer, and fixed amendment concentrations, the results should not be generalized to all agricultural systems. Future studies across different soil types, biochar feedstocks, microplastic characteristics, contamination levels, and field conditions are needed to determine the broader applicability of these findings.

## Data Availability

The datasets presented in this article are not readily available. Requests to access the datasets should be directed to Yanyan Zhang, yanyan.zhang2@mail.mcgill.ca.

## Footnotes

1 http://qiime.org/; https://docs.qiime2.org/2019.4/tutorials/
